# The Microbiology of Acute Exacerbations in Chronic Rhinosinusitis - A Systematic Review

**DOI:** 10.3389/fcimb.2022.858196

**Published:** 2022-03-24

**Authors:** Oghenefejiro Okifo, Amrita Ray, David A. Gudis

**Affiliations:** ^1^ Department of Otolaryngology – Head & Neck Surgery, Henry Ford Health System, Detroit, MI, United States; ^2^ Department of Otolaryngology Head and Neck Surgery, Columbia University, New York City, NY, United States

**Keywords:** microbiology, bacteriology, acute exacerbation, chronic rhinosinusitis, chronic sinusitis, sinus infection

## Abstract

**Background:**

Acute exacerbations (AE) in chronic rhinosinusitis (CRS) are a common and important clinical issue. However, relatively little is known regarding the underlying microbiology that drives exacerbations or how it relates to the microbiome of CRS. The purpose of this study is to examine the literature to characterize the microbiome associated with acute exacerbations in a chronic rhinosinusitis setting. Understanding this disease process may facilitate targeted antibiotic therapy, reduced antibiotic resistance, and offer more effective disease control and treatment efficacy.

**Objective:**

To characterize the microbiome associated with acute exacerbations of chronic rhinosinusitis (AECRS).

**Methods:**

We conducted a systematic review of the literature on Medline, Embase, and Web of Science databases from January 1990-June 2021 to identify studies related to AE in CRS. Exclusion criteria include non-English, non-human studies, and case reports. Studies without culture or PCR data were also excluded.

**Results:**

Fourteen studies were identified which provided detailed data regarding sinus microbiome in AECRS patients. In these patients, a total of 1252 individual isolates were identified. While common acute pathogens were identified in high frequencies in the sinonasal cultures (*Staphylococcus pneumonia, Haemophilus influenza*), the predominant bacteria were *Staphylococcus aureus* (including methicillin-sensitive *Staphylococcus aureus*) and *Pseudomonas aeruginosa.* Patient characteristics that may represent higher risk phenotypes were not consistently collected in the studies. Discussion of antimicrobial sensitivities and/or resistance were included in 7/14 studies.

**Conclusions:**

This systematic review identifies the predominant microbiology species that may contribute to AECRS. Further studies are needed to understand the pathogenic role of bacteria and viruses in AECRS and to identify associated comorbidities and patient phenotypes that may predispose to AE. The optimal treatment regimen for AECRS remains unclear.

## Introduction

Chronic rhinosinusitis (CRS) is an inflammatory disorder of sinonasal cavity that remains one of the leading causes for patients to seek healthcare in the United States (Fokkens et al., 2020). Recent research has begun to characterize the microbiome of normal and diseased sinuses, but our understanding of the role of microbes in CRS remains limited. Mucosal dysbiosis appears to be both a central etiologic factor in the pathogenesis of CRS in addition to a consequence of CRS (Yaniv et al., 2020). Numerous other environmental and host mechanisms have been proposed to drive the pathophysiology of CRS including allergy, ciliary dysfunction, mucosal disruption, immunity derangements, and biofilm formation. Ultimately, patients with CRS tend to experience a course of illness characterized by variable degrees of chronic inflammation with periodic acute exacerbations in symptomology, known as acute exacerbations of chronic rhinosinusitis (AECRS).

Enhanced understanding of the microbiology that contributes to AECRS will facilitate the development of targeted treatment regimens to improve symptoms and disease control, while also reducing the need for inappropriate antibiotic administration and the potential for antibiotic resistance. The purpose of this study is to systematically review the published literature to characterize the underlying microbiology of AECRS.

## Methods

We performed a systematic review utilizing the Preferred Reporting Items for Systematic Reviews and Meta-Analysis (PRISMA) guidelines. A comprehensive search of Medline, Embase, Web of Science, and Google Scholar databases from January 1990-June 2021 was conducted to identify studies relating to the microbiology of acute exacerbations in CRS. A combination of terms was used to maximize the probability of finding all relevant publications, including but not limited to: “rhinitis”, “sinusitis”, “sinus”, “microbiology”, “acute exacerbation”, “chronic disease”, “bacteriology”, “cultures”, and “PCR”.

### Study Selection

Titles and abstracts of all the relevant studies were reviewed by 2 independent authors (OO and AR). Included studies addressed the microbiology of AECRS with either culture or PCR data; studies without culture or PCR data were excluded. Studies were excluded if they were pediatric, non-English, non-human studies, and case reports.

### Data Extraction and Analysis

Data included year of publication, study design, age range, diagnostic criteria, bacterial findings, and immune-histologic findings. After analysis of each article, summary tables were developed. In articles where various data groupings were provided, only the relevant data for the AECRS patient population were extracted and used for analysis.

**Figure 1:**
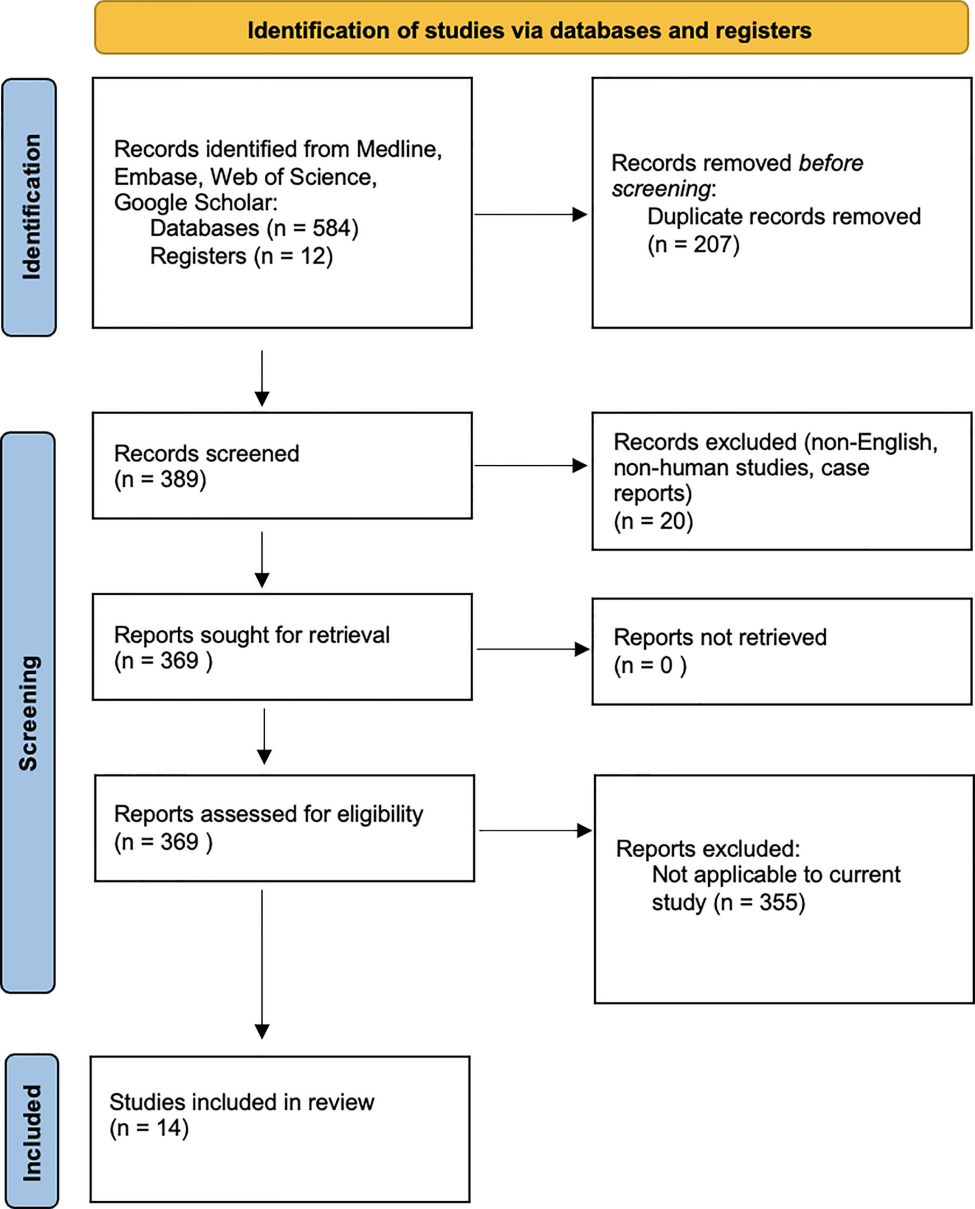
Flow diagram of the study.

A summary of the methods is provided in **Figure 1**.

## Results

### Included Studies

Our initial database search identified 596 articles. Duplicate articles, non-English articles, those without full-text or without extractable data were excluded. A total of 14 articles met the final inclusion criteria for systematic review and underwent further full text review. These studies explored the underlying microbiology in AECRS.

### Microbiology in AECRS

The details of the included studies exploring the microbiology of AECRS are summarized in **Table 1**. The bacteria that were identified with association to AECRS are listed in **Table 2**.

**Table 1:** Descriptive studies of acute microbiology in adult patients with CRS.

Study Author/year	Microbiology present in adult patients with CRS
Matthews BL. et al., 1993 (Matthews et al., 1993)	Clinical trial evaluating cefixime for acute sinusitis or AECRS. N=42
Vaughan WC. Et al. 2002 (Vaughan and Carvalho, 2002)	Cultures obtained in AECRS patients who have undergone prior ESS; examining role of nebulized antibiotics for AECRS. N=42
Namyslowski G. et al., 2002 (Namyslowski et al., 2002)	Clinical trial evaluation of Augmentin and Cefuroxime for AECRS. N=122
Bhattacharyya N. et al., 2004 (Bhattacharyya et al., 2004)	Prospective controlled cohort study. Cultures from pre-op AECRS were compared to post-op ESS. N=17
Brook I, et al., 2005 (Brook et al., 2005)	Aerobic and anaerobic cultures of maxillary sinus secretions. N=7
Brook I. 2006 (Brook, 2006)	Aerobic and anaerobic cultures of CRS and AECRS patients. Similar organism isolated from both patient groups. N=30
Cincik H, et al., 2006 (Cincik and Ferguson, 2006)	Cultures of patients with CRS and AECRS; serial cultures performed. N=27
Coffey CS. et al., 2006 (Coffey et al., 2006)	Cultures of patients with AECRS. Did look at drug resistance. N=77
Ikeda K. et al., 2011 (Ikeda et al., 2011)	Cultures of patients with AECRS and asthma, s/p ESS. N=42
Jiang ZY. et al., 2015 (Jiang et al., 2015)	Retrospective review to examine role of endoscopically driven antibiotic therapy on patient symptoms and endoscopy findings. N=105
Yan CH. et al., 2018 (Yan et al., 2018)	Examined role of culture directed (N=61) vs non-directed (N=61) antibiotics in AECRS.
Vandelaar LJ. et al., 2019 (Vandelaar et al., 2019)	Cultures of CRSwNP, CRSsNP and AFS patients during AECCRS. N=134
Szaleniec J. et al., 2019 (Szaleniec et al., 2019)	Cultures of patients with AECRS, s/p ESS. Did look at drug resistance, and bacteriophage susceptibility. N=50
Yaniv D. et al., 2020 (Yaniv et al., 2020)	Retrospective review of AECRS patients and how bacterial isolates change over time. Did look at drug resistance. N=112

**Table 2:** Microbiology in AECRS infections.

Number of isolates	Organism growth
**258**	S. Aureus
**168**	Pseudomonas aeruginosa
**133**	Haemophilus influenzae
**126**	Methicillin-sensitive S. Aureus
**98**	Streptococcus pneumoniae
**60**	Coag negative staphylococci
**47**	Methicillin-resistant S. Aureus
**31**	Citrobacter diversus
**30**	Escherichia coli
**28**	Staphylococcus epidermidis
**25**	Klebsiella pneumoniae
**22**	Stenotrophomonas maltophilia
**20**	Corynebacterium sp
**19**	Moraxella catarrhalis
**17**	A-hemolytic strep
**17**	Enterobacter sp
**17**	Proteus mirabilis
**16**	Diphtheroids
**14**	Peptostreptococcus species
**9**	Klebsiella oxytoca
**8**	Streptococcus pyogenes
**8**	Acinetobacter sp
**8**	Serratia marcescens
**7**	Streptococcus Group G
**6**	Oral flora (unspecified)
**6**	Acinetobacter
**6**	Moraxella sp
**5**	Citrobacter sp
**4**	Pseudomonas Stutzeri
**4**	B-hemolytic strep
**4**	Microaerophilic streptococci
**4**	Strep agalactiae
**4**	Haem. Parainfluenza
**4**	Citrobacter koseri
**4**	Serratia sp
**3**	Bacteroides species % of its
**2**	Citrobacter freundii
**2**	Xanthomonas sp
**1**	Enterobacter aerogenes
**1**	Enterobacter gergociae
**1**	Alcaligenes fecalis
**1**	Archromobacter sp
**1**	Bacillus sp
**1**	Gemella morbillroum
**1**	Moganella morganii
**1**	Providencia rettgeri

There was significant diversity in the bacteria that were associated with AECRS. The aerobic bacteria included: *Staphylococci* species which included both coagulase-negative *Staph* species and methicillin-resistant *Staph* species, *Streptococcus* species, *Haemophilis influenzae*, *Pseudomonas aeruginosa, Enterobacteriaceae*, and *Moraxella catarrhalis*. The predominant anaerobic bacteria that were identified included: *Prevotella, Porphyromonas, Fusobacterium, Peptostreptococcus*, and *Propionibacterium acnes*, although only limited studies specifically tested or commented on anaerobic growth (Matthews et al., 1993; Vaughan and Carvalho, 2002; Brook et al., 2005; Brook, 2006). Facultative bacteria included the *Escherichia* species and *Klebsiella pneumonia*. The *Staphylococci* species were the most frequently identified culture-positive bacteria.

While most of the studies utilized culture data, one study did include speciation *via* polymerase chain reduction in addition to standard culture alone (Vandelaar et al., 2019). All included studies also commented on aerobic bacterial growth, but anaerobic growth was not routinely reported.

Discussion regarding antibiotic therapy, resistance, and sensitivities was noted in 7 of the 14 studies listed (**Table 3**), although the extent of analysis varied widely by study.

**Table 3:** Key points mentioned regarding antibiotic resistance in key studies.

	Commentary regarding Antibiotic resistance
Matthews et al., 1993	• Looked purely at resistance or susceptibility to cefixime and amoxicillin only • 80% of isolates were susceptible to cefixime, 65% susceptible to amoxicillin
Brook et al., 2005	• Out of 7 patients, 5 were noted to developed antibiotic resistance through B lactamase production • Noted instance of S *pneumoniae* resistance to penicillin
Brook, 2006	• 40% of isolates in AECRS patients developed antibiotic resistance through B lactamase production versus 26% of CRS patients • S *pneumoniae* in AECRS patients were also found to have higher rates of penicillin resistance compared to 0% in CRS patients
Coffey et al., 2006	• Notes that lab did not routinely check for resistance for many of the microbes cultured • In *S. aureus* and *Pseudomonas* species drug resistance was present in 10/48 (21%) and 16/20 (80%), respectively
Ikeda et al., 2011	• Susceptibility tests for S. pneumonia, MRSA, P. aeruginosa, and H. influenzae done on 35 isolates (**Table 1**) against Ampicillin, Methicillin, Cefotaxime, Cefoperazone/sulbactam, Gentamicin, Minomycin, and Levofloxacin ○ Levofloxacin showed excellent efficacy against S. pneumoniae. ○ MRSA was remarkably resistant to all antibiotics except for minomycin. ○ Two isolates of P. aeruginosa was resistant to ampicillin and the third-generation cephalosporins while levofloxacin showed poor activity against only one isolate. • The third- generation cephalosporin and levofloxacin were sensitive to H. influenzae.
Szaleniec et al., 2019	• Mechanisms of antibiotic resistance were identified in 28% of the isolates Consequently, antibiotic-resistant bacteria were carried by 46% of patients. • High rates of resistance noted to amoxicillin/clavulanate (18% of isolates, 28% patients), macrolides (25% of strains, 42% of patients) and clindamycin (30% of strains, 40% of patients). • Resistance to fluoroquinolones and aminoglycosides was very uncommon (6% of isolates, 10% patients). • All isolates including MRSA were sensitive to linezolid.
Yaniv et al., 2020	• Resistant strains identified were either penicillin-resistant *Pneumococcus* or ciprofloxacin-resistant *Pseudomonas*. • The lowest rates of resistance were noted for fluoroquinolones.

## Discussion

Our review demonstrates significant diversity in the various bacteria that were associated with AECRS. *Staphylococci* species were the most frequently identified bacteria, followed by *Pseudomonas aeruginosa*, *Streptococcal* species, and *Haemophilus influenzae*. Among the *Staphylococcal* species, various subspecies were identified including *S. aureus*, MRSA, and coagulase-negative *Staphylococci*. Of note, Rujanavej et al. demonstrated a substantial rise in MRSA isolates from intranasal cultures since the year 2000 and beyond, underscoring the need to consider MRSA coverage in cases of AECRS (Rujanavej et al., 2013). It is also interesting to note the high prevalence of *Pseudomonas* species; given the *Pseudomonal* ability to produce biofilms and multidrug resistance, these findings underscore the value of targeted, antimicrobial therapy (Bhattacharyya and Kepnes, 1999). Of note, there were multiple studies to indicate that anerobic bacteria are present as well, suggesting that the microbial population in AECRS is a mix of aerobic and anaerobic bacteria.

AECRS likely begins with a common viral upper respiratory infection that progresses into a secondary bacterial infection, potentially in an already dysbiotic setting (Brook et al., 2005; Brook, 2006; Cho et al., 2013; Rowan et al., 2015), followed by return to baseline CRS. An exacerbation may also be characterized by worsening sinonasal symptoms, presence of purulence on nasal endoscopy, and/or endoscopically-derived bacterial cultures (Wu et al., 2019; Wu et al., 2020). However, the specific microbiology of these exacerbations remains poorly understood.

Although positive bacterial cultures are identified in up to 90.9% of patients during acute exacerbations (Ikeda et al., 2011), many of the previously utilized treatment paradigms are largely based on the microbiomes of acute or chronic rhinosinusitis states, rather than the particular dysbiome in AECRS.

Currently, there are no consistent treatment guideline for AECRS, but management usually involves short-term antibiotics and/or nasal corticosteroids. Targeted treatment for AECRS requires a better understanding of its pathophysiology. Despite being poorly understood, several factors have been noted to drive this dysbiosis including mucosal inflammation, impaired mucociliary clearance, biofilm formation, chronic mucosal disruption, atrophic rhinitis, transient viral infections and immunologic changes, and arising antibiotic resistance (Lee et al., 2018). Colonization by opportunistic pathogens such as *S. aureus* and *Pseudomonas aeruginosa* have been shown to trigger inflammation that is worsened by defects in the innate immune response.

There is significant evidence that alterations of the sinonasal microbiome are a direct driver of CRS inflammation and acute exacerbations (Rank et al., 2013; Divekar et al., 2015). While not a specific focus of this study, it should be noted that while antibiotic sensitivities were not routinely obtained in all of the included studies, significant multidrug resistance was reported. Thus, there is a growing body of literature to support culture-directed antibiotics to address microbiome shifts that are likely contributing significantly to the underlying disease process.

These intermittent and persistent disorders of the upper airway (including but not limited to asthma, allergic rhinitis, bronchitis, etc.) may represent gradients along a spectrum rather than each being a distinct pathology. In this unified airway theme, inflammatory disruptions in one subsite may affect the homeostasis in others. While this has been studied primarily in allergic disease, less is known about the impact of other adjunct upper airway disorders. For example, new evidence suggests that nasal hyperreactivity to nonspecific allergens may trigger symptoms mimicking AECRS, confounding the clinical picture (Doulaptsi et al., 2020). Additionally, in recent years, the concept of severe chronic upper airway disease (SCUAD) has been proposed to define patients with CRS (with or without polyps) and allergic, nonallergic or occupational rhinitis, whose symptoms are refractory to traditional guideline based treatments. It is worth considering whether these patients represent a group of SCUAD patients, and if so, how to best address the multifactorial underlying etiologies driving the clinical worsening of symptoms (Prokopakis et al., 2014). Thus, cultures obtained during such episodes may not necessarily reflect a true microbiome picture of pure AECRS.

This concept of microbiome shifts during acute exacerbations also mirrors findings from other unified airway subsites. For example, sputum analysis done during acute exacerbations of both chronic obstructive pulmonary disease and chronic bronchitis demonstrate dysbiosis findings similar to those observed in the paranasal sinuses (Dickson et al., 2014; Jubinville et al., 2018). Elevated IL-6 levels have also been linked to patients suffering a CRS exacerbation, suggesting either a viral infection or an altered IL-6 pathway (Yaniv et al., 2020). More robust studies on pathophysiology and treatment options, including randomized controlled trials, are needed to better understand AECRS.

In addition, the articles reviewed included patients at various stages of intervention or recent antimicrobial treatment. It is also worth noting that patients with AECRS are known to have higher prevalence of comorbid conditions including allergic rhinitis, asthma, autoimmune, or other atopic diseases (Kwah et al., 2020). Most of the included studies lacked comprehensive demographic data regarding these and other relevant comorbidities such as respiratory pathologies, diabetes, or extensive obstructive polyposis. Additionally, no study mentioned the role of an odontogenic etiology driving the patient’s CRS, which is starting to become recognized as more prevalent than previously thought (Craig et al., 2021). Thus, characterizing this subtype of CRS requires a more thoughtful and comprehensive approach to identify high risk phenotypes and incorporate preventative measures to reduce exacerbation frequency (Kuiper et al., 2018).

It is worthwhile to consider that traditional culture methods may not adequately depict the *in vivo* polymicrobial host community, as standard cultures offer limited, predefined conditions in which microbial growth can occur. Thus, *in vitro* cultures may inadvertently bias growth to select for faster growing organisms, or those without niche or symbiotic growth needs. Additionally, standard cultures may not adequately represent the microbiome due to physical limitations in obtaining the culture; for example, Miller and Davis demonstrate significant variability in pathogens in cultures from the same patient when done *via* standard methods compared to those obtained intraoperatively (Feazel et al., 2011; Hauser et al., 2015; Miller and Davis, 2018). Thus, it may beneficial to utilize alternative molecular methods of amplification, such as polymerase chain reaction, which can identify up to an order of magnitude more taxa that might be otherwise missed in between 25%-99% of cases. In fact, direct comparisons between sequencing and culture results find that dominant bacteria determined by sequencing is apparent in culture results less than 50% of the time (Feazel et al., 2011).

Although this discussion regarding AECRS focuses primarily on underlying bacterial pathogens, it is important to keep in mind that viruses and fungi may also be drivers of AECRS. However, the literature is limited in understanding the delicate balance of the baseline microbiome or the role of other microbes. Although rhinovirus presence has been identified as being the most prevalent virus in CRS exacerbation in some studies, its mechanism of pathogenesis and relationship to bacterial dysbiosis is unclear (Cho et al., 2013; Yaniv et al., 2020).

There is also limited and conflicting literature to describe the role endoscopic sinus surgery (ESS) may play in altering the sinus microbiome, possibly *via* mechanisms that alter sinonasal aeration, mucociliary clearance, inflammatory profiles, nitric oxide levels, and others. Larson and Han describe their findings in 26 patients, demonstrating that ESS does not significant alter the pre and post-surgery microbiome (Larson and Han, 2011). Hai et al. specifically examined the effect of ESS on biofilm production, finding that although ESS does not completely eradicate biofilms, it does significantly reduce their density (Hai et al., 2010). Several other studies demonstrate worse patient outcomes after ESS where biofilms are involved (Bendouah et al., 2006; Psaltis et al., 2008; Zhang et al., 2009).

The above discussion illustrates the complexity in appropriately identifying and treating AECRS. In addition to diligent and thoughtful characterization of clinical symptoms, advancements in molecular technology are already enabling research in the unique endo- and phenotypes of this disease, and allow for customized, precision treatment, termed “precision medicine” (Vlastos et al., 2019).

## Conclusion

This systematic review identifies the predominant microbiology species that may contribute to AECRS. The literature supports a pathogenic role of bacteria and viruses in AECRS distinct from those cultured at baseline for patients with CRS. The optimal treatment regimen for AECRS remains unclear.

## Author Contributions

OO: data collection, manuscript development. AR: data collection, manuscript development, editing and study design. DG: manuscript development, editing, and study design. All authors contributed to the article and approved the submitted version.

## Conflict of Interest

The authors declare that the research was conducted in the absence of any commercial or financial relationships that could be construed as a potential conflict of interest.

## Publisher’s Note

All claims expressed in this article are solely those of the authors and do not necessarily represent those of their affiliated organizations, or those of the publisher, the editors and the reviewers. Any product that may be evaluated in this article, or claim that may be made by its manufacturer, is not guaranteed or endorsed by the publisher.
